# ﻿Review of the genus *Ageleradix* Xu & Li, 2007 (Araneae, Agelenidae), with descriptions of three new species

**DOI:** 10.3897/zookeys.1236.143650

**Published:** 2025-05-02

**Authors:** Yan-Nan Mu, Lu-Yu Wang, Tian-Yu Ren, Yuan Tian, Zhi-Sheng Zhang

**Affiliations:** 1 Key Laboratory of Eco-environments in Three Gorges Reservoir Region (Ministry of Education), School of Life Sciences, Southwest University, Chongqing 400715, China Southwest University Chongqing China; 2 Yunnan Nangunhe National Nature Reserve Management and Protection Bureau, Lincang, China Yunnan Nangunhe National Nature Reserve Management and Protection Bureau Lincang China

**Keywords:** Ageleninae, Asia, funnel weavers, identification key, morphology, taxonomy

## Abstract

The spider genus *Ageleradix* Xu & Li, 2007 is reviewed. Three new species are described: *A.dulong* Mu, Wang & Zhang, **sp. nov.** (♂♀, Yunnan), *A.jinfoshan* Mu, Wang & Zhang, **sp. nov.** (♀, Chongqing) and *A.nangunhe* Mu, Wang & Zhang, **sp. nov.** (♂♀, Yunnan). A key to all nine species of *Ageleradix* is provided, and the genus is split into three species-groups.

## ﻿Introduction

Agelenidae C.L. Koch, 1837 comprises 1427 species in 98 genera (including 1 extinct genus and 7 species) and is distributed almost worldwide. In China, the family is represented by 490 species, belonging to 39 genera in four subfamilies (Zhu et al. 2017; WSC 2024). *Ageleradix* Xu & Li, 2007 described based on *A.sichuanensis* Xu & Li, 2007, and placed in the subfamily Ageleninae C.L. Koch, 1837 (Zhu et al. 2017) currently comprising six species, all reviewed by Zhu et al. (2017). The genus is distributed in Guangxi, Guizhou, Sichuan, Xizang and Yunnan provinces of China.

While examining specimens collected from Yunnan and Chongqing, three new species of *Ageleradix* were recognized. This paper aims to describe these new species, provide a comprehensive review of the genus, and present an identification key for all its known species and notes on species grouping.

## ﻿Material and methods

All specimens were preserved in 75% ethanol and examined, illustrated, photographed, and measured using a Leica M205A stereomicroscope equipped with a drawing tube, a Leica DFC450 Camera, and LAS v. 4.6 software. Male palps and epigynes were examined and illustrated after they were dissected. Epigynes were cleared by immersing them in pancreatin for about an hour (Álvarez-Padilla and Hormiga 2007). Eye sizes were measured as the maximum diameter. Leg measurements are shown as: total length (femur, patella and tibia, metatarsus, tarsus). All measurements are in millimeters. Specimens examined here are deposited in the Collection of Spiders, School of Life Sciences, Southwest University, Chongqing, China (SWUC).

Terminology follows Xu and Li (2007) and Zhang et al. (2008). Abbreviations used in the text: **ALE** = anterior lateral eye; **AME** = anterior median eye; **PLE** = posterior-lateral eye; **PME** = posterior median eye.

## ﻿Taxonomy

**Family Agelenidae C. L. Koch, 1837**

**Subfamily Ageleninae C. L. Koch, 1837**

### ﻿ Ageleradix

Taxon classificationAnimaliaAraneaeAgelenidae

Xu & Li, 2007

2E88AEF6-C211-5DBA-8C06-08B47CE8ACF5

#### Type species.

*Ageleradixsichuanensis* Xu & Li, 2007 (by original designation).

#### Diagnosis.

This genus is similar to *Allagelena* Zhang, Zhu & Song, 2006 in having centrally originated, extending distally and proximally and sclerotized conductor (C), but can be separated from it by: palpal patella lacking apophysis (vs. with apophysis), retrolateral tibial apophysis (RTA) not well developed (vs. well developed), embolus (E) slender (vs. thick), tegular apophysis (TA) well developed (vs. not well developed); scape (Sc) extending to middle part of epigynal plate (vs. absent or not extending to middle part), and atrium shallow (vs. deep).

#### Composition.

*A.cymbiforma* (Wang, 1991) (♀), *A.otiforma* (Wang, 1991) (♀♂), *A.schwendingeri* Zhang, Li & Xu, 2008 (♀♂), *A.sichuanensis* Xu & Li, 2007 (♀♂), *A.sternseptum* Zhang, Li & Xu, 2008 (♀) and *A.zhishengi* Zhang, Li & Xu, 2008 (♀♂), *A.dulong* Mu, Wang & Zhang sp. nov. (♀♂), *A.jinfo*shan Mu, Wang & Zhang sp. nov. (♀) and *A.nangunhe* Mu, Wang & Zhang sp. nov. (♀♂).

#### Distribution.

Known only from China (Yunnan, Sichuan, Xizang, Guangxi, Guizhou).

### ﻿ Ageleradix dulong

Taxon classificationAnimaliaAraneaeAgelenidae

Mu, Wang & Zhang sp. nov.

737D0EA2-2834-5CE0-901F-4592B3CD4283

https://zoobank.org/65098D5D-3675-4B82-8C02-EE84112EAFAD

**Figure 1.**
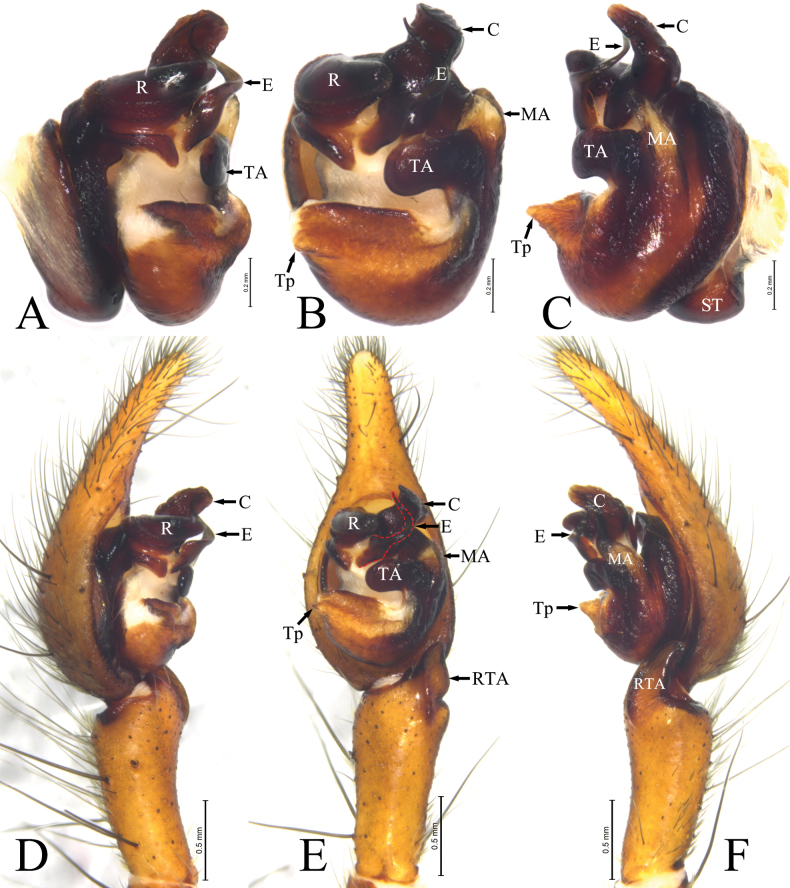
*Ageleradixdulong* Mu, Wang & Zhang, sp. nov., male left palp **A** bulb, prolateral view **B** same, ventral view **C** same, retrolateral view **D** prolateral view **E** ventral view **F** retrolateral view. Abbreviations: **C**—conductor; **E**—embolus; **MA**—median apophysis; **R**—radix; **RTA**—retrolateral tibial apophysis; **TA**—tegular apophysis; **Tp**—tegular process; **ST**—subtegulum.

**Figure 2.**
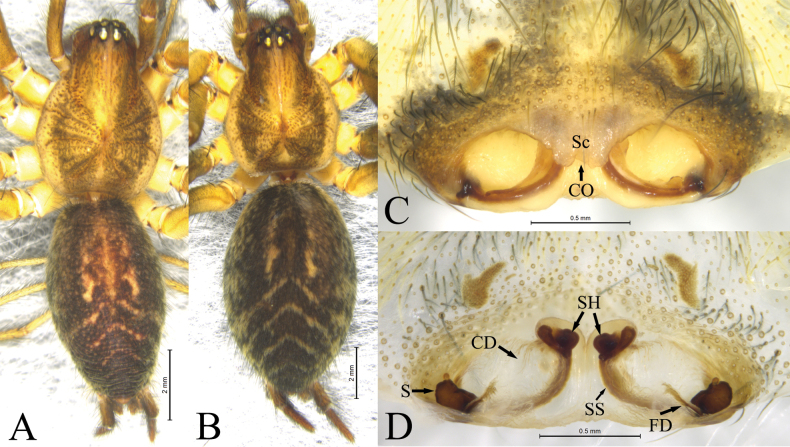
*Ageleradixdulong* Mu, Wang & Zhang, sp. nov. Male holotype (**A**) and female paratype (**B–D**) **A, B** habitus, dorsal view **C** epigyne, ventral view **D** same, dorsal view. Abbreviations: **CD**—copulatory duct; **CO**—copulatory opening; **FD**—fertilization duct; **S**—spermathecae; **Sc**—scape; **SH**—spermathecal head; **SS**—spermathecal stalk.

Chinese name: 独龙盾漏斗蛛 Figs 1 , 2

#### Type material.

***Holotype*** • ♂, China, Yunnan Prov., Nujiang Lisu Auton. Pref., Gongshan Dulong and Nu Auton. Co., Dulong River, dangbanglaka; 27°49'38.85"N, 98°19'35.52"E, elev. 1430 m, 20.04.2024, leg. L.Y. Wang, et al. ***Paratypes***: • 2♂1♀, with same data as holotype. • 1♀, Hapang waterfall; 27°40'43.66"N, 98°16'13.25"E, elev. 1156 m, leg. L.Y. Wang.

#### Etymology.

The specific name is derived from the Dulong River; noun in apposition.

#### Diagnosis.

The new species resembles *A.schwendingeri* in having similar median apophysis (MA) and retrolateral tibial apophysis (RTA) (cf. Fig. 1D–F, Fig. 5E–G, and figs 10–11 in Zhang et al. 2008), but can be differentiated from by: 1) palpal tibia long, about 2/3 length of cymbium (Fig. 1D–F) (vs. short, about 1/4 length of cymbium, Fig. 5G and fig. 10–12 in Zhang et al. 2008); 2) embolus (E) with wide base and curved tip (Fig. 1D–F) (vs. thin base and straight tip, Fig. 5F and fig. 11 in Zhang et al. 2008); 3) conductor (C) tongue-shaped with narrow tip (Fig. 1D–F) (vs. not tongue-shaped, with wider tip, Fig. 5F, G and fig. 12 in Zhang et al. 2008); 4) scape (Sc) extending posteriorly to the center of atrium (Fig. 2C) (vs. scape extending to posterior edge of atrium, Fig. 5C and fig. 14 in Zhang et al. 2008); and 5) spermathecae (S) kidney-shaped (Fig. 2D) (vs. spherical, Fig. 5D fig. 16 in Zhang et al. 2008).

#### Description.

**Male** (holotype, Fig. 2A). Total length 10.91. Carapace: 4.94 long, 3.60 wide. Abdomen: 5.83 long, 3.28 wide. Eye sizes and interdistances: AME 0.28, ALE 0.32, PME 0.25, PLE 0.30, AME–AME 0.09, AME–ALE 0.07, PME–PME 0.17, PME–PLE 0.16, ALE–PLE 0.07. MOA: anterior width 0.59, posterior width 0.67, 0.70 long. Clypeus 0.37 long. Chelicerae with 3 promarginal and 3 retromarginal teeth. Leg measurements: I 28.48 (7.65, 9.13, 8.04, 3.66), II 24.64 (6.81, 7.61, 6.78, 3.44), III 21.29 (5.89, 6.23, 6.18, 2.99), IV 25.88 (6.67, 7.60, 8.03, 3.58). Carapace yellow, with U-shaped brown pattern. Cervical groove and radial groove distinct. Fovea short, slightly depressed. Abdomen ovoid, gray; cardiac mark red-brown; posterodorsal part of abdomen with 3 distinct chevrons. Anterior spinnerets shorter than basal segment of posterior-lateral spinnerets.

Palp (Fig. 1A–F). Tibia about 2/3 length of cymbium, tibial apophysis (RTA) nubbly, extending towards dorsal part of cymbium. Cymbial tip long, about half length of cymbium. Bulb oval, about half length of cymbium. Tegulum with cuspides process (Tp) at middle of prolateral margin. Tegular apophysis (TA) transverse, tip round. Conductor (C) lamellar, heavily sclerotized, tongue-shaped, tip curved toward prolateral, with several sclerites at retro-surface. Radix (R) strongly sclerotized, tip blunt. Median apophysis (MA) straight, formed concavity in ventral view, with blunt end. Embolus (E) originating from anterior part of tegulum, hook-shaped in ventral view and S-shaped in prolateral view, tapering from base to tip.

**Female** (paratype, Fig. 2B). Total length 14.34. Carapace: 5.74 long, 4.46 wide. Abdomen: 8.29 long, 5.22 wide. Eye sizes and interdistances: AME 0.34, ALE 0.37, PME 0.31, PLE 0.36, AME–AME 0.12, AME–ALE 0.09, PME–PME 0.24, PME–PLE 0.21, ALE–PLE 0.10. MOA: anterior width 0.72, posterior width 0.82, 0.87 long. Clypeus 0.49 long. Leg measurements: I 23.52 (6.47, 7.69, 5.93, 3.43), II 20.65 (5.83, 6.58, 5.19, 3.05), III 18.55 (5.43, 5.61, 4.98, 2.53), IV 23.91 (6.65, 7.51, 6.73, 3.02). All other somatic characters same as in male.

Epigyne (Fig. 2C, D). Atrium oval, located posteriorly, binocular-shaped, anterior edge of atrium with kind of scape (Sc). Copulatory openings (CO) located anteromesally. Copulatory ducts (CD) transparent, membranous, “八” shaped. Spermathecae (S) kidney-shaped, located at posterior-lateral edge epigynal plate, spermathecal stalk (SS) tube-like, spermathecal head (SH) oval, anteriorly located in dorsal view. Fertilization ducts (FD) extending anteromesally.

#### Distribution.

Known only from the type locality.

### ﻿ Ageleradix jinfoshan

Taxon classificationAnimaliaAraneaeAgelenidae

Mu, Wang & Zhang sp. nov.

00B65A4C-2CFC-5D9A-8C0E-8B1E229D5C18

https://zoobank.org/025747BE-8F6A-40DB-89A3-875AFC819008

**Figure 3.**
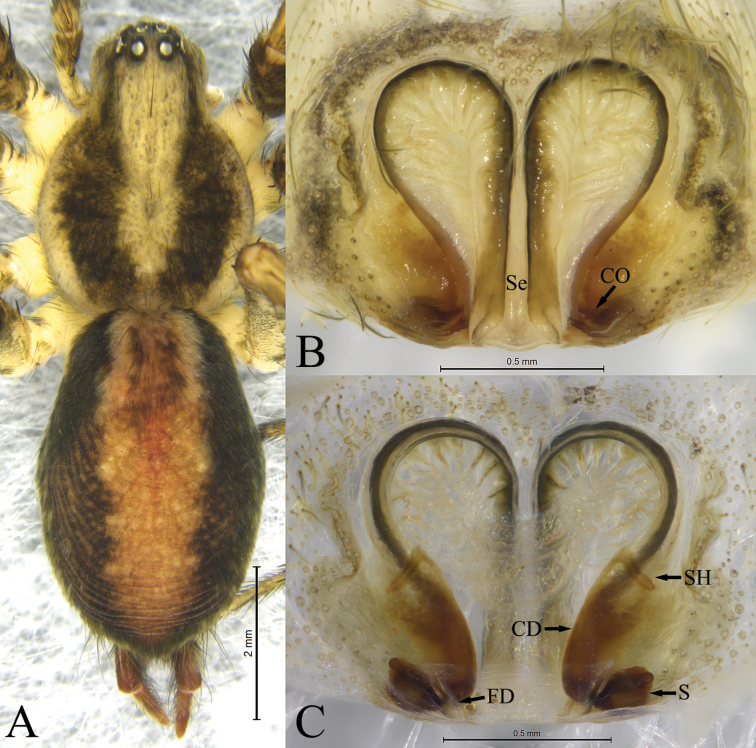
*Ageleradixjinfoshan* Mu, Wang & Zhang, sp. nov., female holotype **A** dorsal view **B** epigyne, ventral view **C** same, dorsal view. Abbreviations: **CD**—copulatory duct; **CO**—copulatory opening; **FD**—fertilization duct; **S**—spermathecae; **Se**—septum; **SH**—spermatheacl head.

Chinese name: 金佛山漏斗蛛 Fig. 3

#### Type material.

***Holotype*** • ♀, China, Chongqing Mun., Nanchuan Dist., Jinfo Mt Reserve (Gufo Cave); 29°2'6.93"N, 107°11'32.14"E, elev. 2043 m, 4.09.2024, leg. Z.S. Zhang.

#### Etymology.

The specific name is derived from the type locality (jinfoshan = Jinfo mountain); noun in apposition.

#### Diagnosis.

The new species resembles *A.zhishengi* in having similar-shaped anterior part of atrium (cf. Fig. 3B, C, and fig. 29A, B in Zhu et al. 2017), but can be differentiated from it by: 1) septum (Se) reaching posterior edge of epigyne (Fig. 3B) (vs. epigyne with scape, fig. 29A in Zhu et al. 2017), and 2) copulatory duct (CD) nearly straight (Fig. 3C) (vs. strongly curved, fig. 29B in Zhu et al. 2017).

#### Description.

**Female**. Total length 7.94. Carapace: 3.68 long, 2.91 wide. Abdomen: 4.70 long, 2.95 wide. Eye sizes and interdistances: AME 0.22, ALE 0.24, PME 0.20, PLE 0.21, AME–AME 0.07, AME–ALE 0.06, PME–PME 0.19, PME–PLE 0.12, ALE–PLE 0.07. MOA: anterior width 0.44, posterior width 0.55, 0.48 long. Clypeus 0.10 long. Chelicerae with 3 promarginal and 3 retromarginal teeth. Leg measurements: I 11.84 (3.18, 4.07, 2.73, 1.86), II 10.26 (3.00, 3.29, 2.33, 1.64), III 10.19 (2.79, 3.29, 2.57, 1.54), IV 13.96 (3.83, 4.32, 3.81, 2.00). Carapace white-yellow, with U-shaped dark brown pattern. Cervical groove and radial grooves distinct. Fovea short, slightly depressed. Abdomen ovoid, gray; cardiac mark nearly as long as abdomen, red-brown. Anterior spinnerets shorter than basal segment of posterior-lateral spinnerets.

Epigyne as in Fig. 3B, C. Atrium balloon-shaped, membranous, with distinct septum (Se) reaching posterior margin, more than 3 times longer than wide, with parallel margins. Copulatory opening (CO) located posteriorly. Copulatory ducts (CD) curved. Spermathecal head (SH) clavate. Spermathecae (S) fist-shaped, posteriorly located spaced by about 2 times diameter of spermathecae. Fertilization ducts (FD) extending antero-laterally.

**Male.** Unknown.

#### Distribution.

Known only from the type locality.

### ﻿ Ageleradix nangunhe

Taxon classificationAnimaliaAraneaeAgelenidae

Mu, Wang & Zhang sp. nov.

5B913D7C-CCBF-5CC2-8054-B389B02A6D97

https://zoobank.org/0CD4166C-E283-4EF3-B3E4-181EE2084F2F

**Figure 4.**
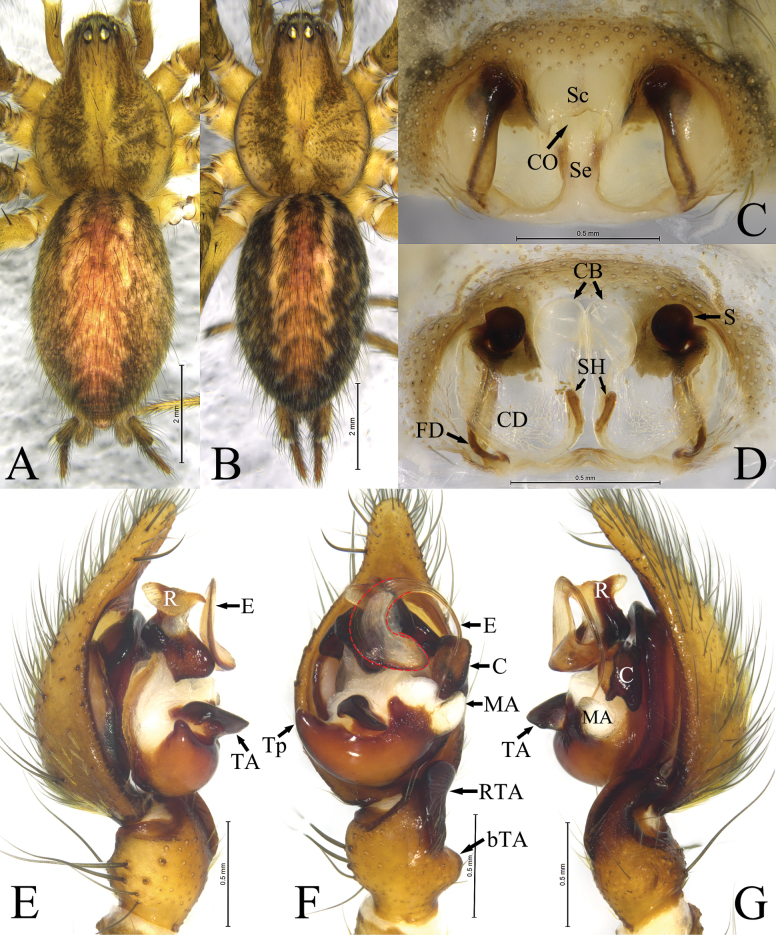
Mu, Wang & Zhang, sp. nov., male holotype and female paratype **A** male, dorsal view **B** female, dorsal view **C** epigyne, ventral view **D** same, dorsal view **E** left palp, prolateral view **F** same, ventral view **G** same, retrolateral view. Abbreviations: **bTA**—basal tibial apophysis; **C**—conductor; **CB**—copulatory bursa; **CD**—copulatory duct; **CO**—copulatory opening; **E**—embolus; **FD**—fertilization duct; **MA**—median apophysis; **R**—radix; **RTA**—retrolateral tibial apophysis; **S**—spermathecae; **Sc**—scape; **Se**—septum; **SH**—spermathecal head; **TA**—tegular apophysis; **Tp**—tegular process.

Chinese name: 南滚河盾漏斗蛛 Fig. 4

#### Type material.

***Holotype*** • ♂, China, Yunnan Prov., Lincang City, Cangyuan Co., Nangunhe National Nature Reserve, Mengjiao station; 23°16'36.01"N, 99°11'24.13"E, elev. 1747 m, 29.09.2024, leg. Y.J. Cai and L.X. Cheng. ***Paratypes***: • 2♀, with same data as holotype.

#### Etymology.

The specific name is derived from the type locality; noun in apposition.

#### Diagnosis.

**Figure 5.**
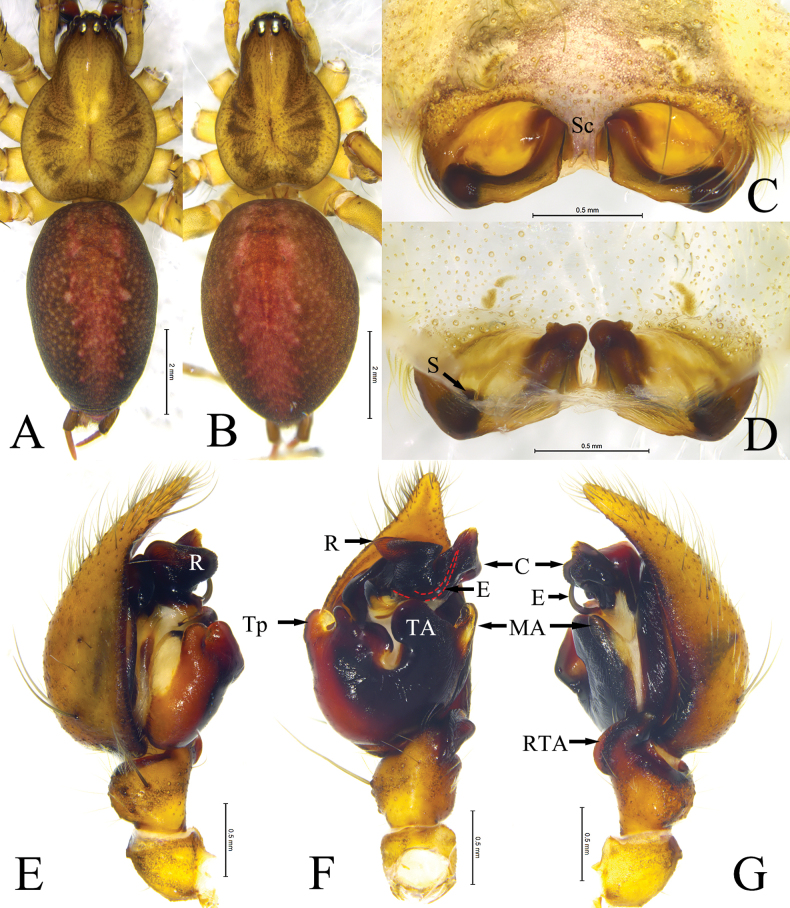
Habitus of *Ageleradixschwendingeri* Zhang, Li & Xu, 2008 **A** male, dorsal view **B** female, dorsal view **C** epigyne, ventral view **D** same, dorsal view **E** male left palp, prolateral view **F** same, ventral view **G** same, retrolateral view. Abbreviations: **C**—conductor; **E**—embolus; **MA**—median apophysis; **R**—radix; **RTA**—retrolateral tibial apophysis; **TA**—tegular apophysis; **Tp**—tegular process; **S**—spermathecae; **Sc**—scape.

The male of this new species resembles those of *A.schwendingeri* in having similarly shaped retrolateral tibial apophysis (RTA) and short tibia (cf. Fig. 4E–G and Fig. 5E–G, fig. 26D in Zhu et al. 2017), but can be differentiated from it by: 1) embolus (E) long, filiform (Fig. 4E–G) (vs. short, hooked, fig. 26D in Zhu et al. 2017); and 2) conductor (C) membranous, with rounded tip (Fig. 4E–G) (vs. strongly sclerotized, tip winding, Fig. 4E–G and Fig. 5E–G). The female of the new species resembles those of *A.cymbiforma* in having similarly shaped atrium located posteriorly (cf. Fig. 4C and fig. 24A in Zhu et al. 2017), but can be differentiated from it by: 1) septum (Sp) 2 times longer than wide (Fig. 4C) (vs. narrow, more than 4 times longer than wide, fig. 24A in Zhu et al. 2017); and 2) copulatory bursae (CB) balloon-shaped (Fig. 4D) (vs. clavate, fig. 24B in Zhu et al. 2017).

#### Description.

**Male** (holotype, Fig. 4A). Total length 8.40. Carapace: 3.90 long, 2.82 wide. Abdomen: 4.90 long, 2.86 wide. Eye sizes and interdistances: AME 0.23, ALE 0.25, PME 0.19, PLE 0.25, AME–AME 0.08, AME–ALE 0.09, PME–PME 0.16, PME–PLE 0.13, ALE–PLE 0.11. MOA: anterior width 0.49, posterior width 0.56, 0.58 long. Clypeus 0.19 long. Chelicerae with 3 promarginal and 5 retromarginal teeth. Leg measurements: I 16.77 (4.37, 5.35, 4.42, 2.63), II 13.74 (3.80, 4.20, 3.50, 2.24), III 12.68 (3.46, 3.66, 3.64, 1.92), IV 17.07 (4.55, 5.08, 5.20, 2.24). Carapace yellow, with 2 rows of brown stripe. Cervical groove and radial groove distinct. Fovea short, slightly depressed. Abdomen ovoid, gray; cardiac mark red-brown. Anterior spinnerets shorter than basal segment of posterior-lateral spinnerets.

Palp (Fig. 4E–G). Tibia shorter than cymbium, about 1/3 length of cymbium, with small retrolateral lump (bTA). Retrolateral tibial apophysis (RTA) nearly as long as tibia, nubbly, roundly bent dorsally. Cymbial tip about 1/5 width of median part of cymbium. Bulb oval. Tegulum with horseshoe-shaped apophysis (TA) located almost medially, retrolateral margin with subconical baso-prolateral process (Tp). Conductor (C) nubbly, extending posteriorly, bifurcated at tip. Radix (R) with sclerotized base and membranous, tongue-shaped tip. Median apophysis (MA) membranous, thumb-shaped. Embolus (E), with wide base, gradually tapering into filamentous embolus proper, roundly clockwise bent embolus proper as long as half of bulb, tip resting on 3'clock position.

**Female** (paratype Fig. 4B). Total length 9.18. Carapace: 4.29 long, 3.17 wide. Abdomen: 5.28 long, 2.96 wide. Eye sizes and interdistances: AME 0.26, ALE 0.26, PME 0.23, PLE 0.25, AME–AME 0.09, AME–ALE 0.10, PME–PME 0.18, PME–PLE 0.14, ALE–PLE 0.11. MOA: anterior width 0.51, posterior width 0.59, 0.62 long. Clypeus 0.15 long. Chelicerae with 3 promarginal and 4 retromarginal teeth. Leg measurements: I 15.19 (4.06, 4.92, 3.74, 2.47), II 12.98 (3.66, 4.15, 3.21, 1.96), III 12.83 (3.56, 4.08, 3.34, 1.85), IV 17.42 (4.81, 5.21, 5.11, 2.29). Color darker than male, appearance of body similar as male.

Epigyne (Fig. 4C–D). Atrium large, posteriorly located, with distinct scape (Sc) and septum (Se). Copulatory openings (CO) located anteromesally. Copulatory ducts (CD) transparent, membranous, sclerotiozed part encircling spermatecae. Copulatory bursae (CB) balloon-shaped, transparent. Spermathecae (S) spherical, located anteriorly. Spermathecal head (SH) clavate. Fertilization ducts (FD) long, well sclerotized extending to posterior of epigynal plate.

#### Distribution.

Known only from the type locality.

### ﻿ Ageleradix schwendingeri

Taxon classificationAnimaliaAraneaeAgelenidae

Zhang, Li & Xu, 2008

450E6290-141D-5CB4-AC13-A730885F6C9F

Fig. 5

#### Material examined.

**China, Xizang** • 2♂3♀, Chayu Co., Xiachayu Town, Xiachayu Bridge, scrub-grassland near river, 28°27'24.72"N, 97°02'40.68"E, elev. 1464 m, 26.06.2018, leg. L.Y. Wang et al. • 1♀, Chayu Co., 28°39'35.88"N, 97°27'57.84"E, elev. 2323 m, 25.06.2018, leg. L.Y. Wang. • 1♂1♀, Chayu Co., 28°39'35.88"N, 97°27'57.84"E, elev. 2323 m, 25.05.2019, leg. L.Y. Wang and P. Liu. • 1♂, Chayu Co., 28°39'35.88"N, 97°27'57.84"E, elev. 2323 m, 27.05.2019, leg. L.Y. Wang.

#### Diagnosis and description.

See Zhu et al. (2017).

#### Distribution.

China (Xizang, Sichuan).

### ﻿Key to species of *Ageleradix*

**Table:** 

1	Female	**2**
–	Male	**10**
2	Atrium posteriorly located	**3**
–	Atrium anteriorly located	**6**
3.	Spermathecae (S) anteriorly located	**4**
–	Spermathecae (S) posteriorly located	**5**
4	Copulatory bursa (CB) oval	***A.nangunhe* sp. nov.**
–	Copulatory bursa (CB) clavate	***A.cymbiforma***
5	Scape (Sc), bifurcated with blunt tips wider than long	***A.dulong* sp. nov.**
–	Scape (Sc), bifurcated with pointed tips longer than wide	***A.schwendingeri***
6	Scape (Sc) lacking	**7**
–	Scape (Sc) present	**8**
7	Atrium divided	***A.sternseptum***
–	Atrium undivided	***A.otiforma***
8	Spermathecae (S) round	***A.sichuanensis***
–	Spermathecae (S) oval	**9**
9	Septum (Se) lacking	***A.zhishengi***
–	Septum (Se) as long as atrium, with parallel margins	***A.jinfoshan* sp. nov.**
10	RTA developed, large; conductor (C) tongue-shaped	**11**
–	RTA strongly reduced or absent; conductor (C) not tongue-shaped	**13**
11	Embolus (E) reach mid part of bulb, filiform	***A.nangunhe* sp. nov.**
–	Embolus (E) extending anteriorly	**12**
12	Tibia about 2/3 length of cymbium, embolus (E) S-shaped in retrolateral view	***A.dulong* sp. nov.**
–	Tibia about 1/4 length of cymbium, embolus (E) C-shaped in retrolateral view	***A.schwendingeri***
13	Embolus (E) long, filiform bent anticlockwise	***A.otiforma***
–	Embolus (E) short	**14**
14	Conductor (C) longer than bulb, tibia wider than long	***A.zhishengi***
–	Conductor (C) about1/2 of bulb length, tibia longer than wide	***A.sichuanensis***

## ﻿Discussion

The genus *Ageleradix* now comprises nine species, all distributed in southwest China. Although species within this genus appear similar in general appearance, they exhibit significant differences in copulatory organs. For instance, in *A.zhishengi*, the conductor is longer than the bulb, whereas in other species, it is shorter than the bulb. In addition, *A.otiforma* also display notable differences compared to the type species *A.sichuanensis*. For example, *A.otiforma* exhibits a filiform embolus, a conductor with a membranous tip, and the scape absent in the epigyne, while in contrast, *A.sichuanensis* has a short embolus, sclerotized conductor, and the scape extends to the middle part of the epigynal plate.

In summary, we propose that *Ageleradix* can be divided into three species-groups based on the shape of copulatory organs: the *A.cymbiforma*-group, the *A.otiforma*-group, and the *A.sichuanensis*-group. The diagnosis and composition of each species group are provided in Table 1.

**Table 1.** Characteristics of the *Ageleradix* species-groups and list of species in each.

Species group name	Diagnostic character	Species
*A.cymbiforma*-group	RTA well developed, nubbly	*A.cymbiforma*; *A.dulong* sp. nov.; *A.nangunhe* sp. nov.; *A.schwendingeri*
	conductor tongue-shaped	
	atrium posteriorly located; with scape	
*A.otiforma*-group	RTA with sharp tip	*A.otiforma* (Wang, 1991); *A.sternseptum* Zhang, Li & Xu, 2008
	conductor long, with narrow and membranous tip	
	atrium anteriorly located; scape absent	
*A.sichuanensis*-group	RTA inconspicuous	*A.sichuanensis* Xu & Li, 2007; *A.zhishengi* Zhang, Li & Xu, 2008; *A.jinfoshan* sp. nov.
	conductor strong sclerotized, nubbly	
	atrium large, anteriorly located; scape present	

## Supplementary Material

XML Treatment for Ageleradix

XML Treatment for Ageleradix dulong

XML Treatment for Ageleradix jinfoshan

XML Treatment for Ageleradix nangunhe

XML Treatment for Ageleradix schwendingeri

## ﻿Additional information

### Author ORCIDs

Yan-Nan Mu https://orcid.org/0000-0002-2504-673X

Luyu Wang https://orcid.org/0000-0002-5250-3473

Yuan Tian https://orcid.org/0009-0007-5358-271X

Zhi-Sheng Zhang https://orcid.org/0000-0002-9304-1789

### Data availability

All of the data that support the findings of this study are available in the main text.
